# Effects of Combined Balance and Strength Training on Measures of Balance and Muscle Strength in Older Women With a History of Falls

**DOI:** 10.3389/fphys.2020.619016

**Published:** 2020-12-23

**Authors:** Sghaier Zouita, Hassane Zouhal, Habiba Ferchichi, Thierry Paillard, Catherine Dziri, Anthony C. Hackney, Ismail Laher, Urs Granacher, Amira Ben Moussa Zouita

**Affiliations:** ^1^Higher Institute of Sport and Physical Education, Ksar-said, University of Manouba, Manouba, Tunisia; ^2^M2S (Laboratoire Mouvement, Sport, Santé), University of Rennes, Rennes, France; ^3^Department of Medicine Physical and Functional Rehabilitation of the National Institute of Orthopedics “M.T. Kassab”, Tunis, Tunisia; ^4^Movement, Balance, Performance and Health Laboratory, Tarbes, E2S/University of Pau and Pays de l’Adour, Pau, France; ^5^Department of Exercise and Sport Science, Department of Nutrition, University of North Carolina at Chapel Hill, Chapel Hill, NC, United States; ^6^ Department of Anesthesiology, Pharmacology and Therapeutics, University of British Columbia, Vancouver, BC, Canada; ^7^Division of Training and Movement Science, University of Potsdam, Potsdam, Germany

**Keywords:** aging, exercise, postural sway, force, tasks

## Abstract

**Objective:**

We investigated the effects of combined balance and strength training on measures of balance and muscle strength in older women with a history of falls.

**Methods:**

Twenty-seven older women aged 70.4 ± 4.1 years (age range: 65 to 75 years) were randomly allocated to either an intervention (IG, *n* = 12) or an active control (CG, *n* = 15) group. The IG completed 8 weeks combined balance and strength training program with three sessions per week including visual biofeedback using force plates. The CG received physical therapy and gait training at a rehabilitation center. Training volumes were similar between the groups. Pre and post training, tests were applied for the assessment of muscle strength (weight-bearing squat [WBS] by measuring the percentage of body mass borne by each leg at different knee flexions [0°, 30°, 60°, and 90°], sit-to-stand test [STS]), and balance. Balance tests used the modified clinical test of sensory interaction (mCTSIB) with eyes closed (EC) and opened (EO), on stable (firm) and unstable (foam) surfaces as well as spatial parameters of gait such as step width and length (cm) and walking speed (cm/s).

**Results:**

Significant group × time interactions were found for different degrees of knee flexion during WBS (0.0001 < *p* < 0.013, 0.441 < *d* < 0.762). *Post hoc* tests revealed significant pre-to-post improvements for both legs and for all degrees of flexion (0.0001 < *p* < 0.002, 0.697 < *d* < 1.875) for IG compared to CG. Significant group × time interactions were found for firm EO, foam EO, firm EC, and foam EC (0.006 < *p* < 0.029; 0.302 < *d* < 0.518). *Post hoc* tests showed significant pre-to-post improvements for both legs and for all degrees of oscillations (0.0001 < *p* < 0.004, 0.753 < *d* < 2.097) for IG compared to CG. This study indicates that combined balance and strength training improved percentage distribution of body weight between legs at different conditions of knee flexion (0°, 30°, 60°, and 90°) and also decreased the sway oscillation on a firm surface with eyes closed, and on foam surface (with eyes opened or closed) in the IG.

**Conclusion:**

The higher positive effects of training seen in standing balance tests, compared with dynamic tests, suggests that balance training exercises including lateral, forward, and backward exercises improved static balance to a greater extent in older women.

## Introduction

Falls are a major cause of unintentional injury worldwide (Caldevilla et al., 2013). For people aged 65 or older, falls are the most common accident and can result in death, hospitalization, disability, loss of independence. In addition, fear of falling can lead to activity restriction and reduce daily functions (Baixinho et al., 2014). The economic costs of falls related to medical and social care are substantial (Herdman and Kamitsuru, 2014). Risk factors for falls include environmental factors such as poor lightning, or internal factors such as balance deficits, gait disorders, and muscle weakness (Iglesias et al., 2017). Fall history, medication use, and deficits in mobility, muscle strength, gait and balance, have frequently been listed as fall risk factors (Baixinho et al., 2014; Sousa et al., 2016). In addition to chronic diseases, falls constitute another health care burden that is associated with high treatment costs and reduced quality of life (Hortobágyi et al., 2020).

There is an age-related loss in the ability of sensory (vestibular, visual, and somatosensory), cognitive (central nervous system), and musculoskeletal systems to control balance (Dunsky, 2019), causing the elderly to rely more on visual information. Visual feedback delays affected postural corrections in older adults, with larger increases in postural sway, suggesting that older adults prioritize vision to control posture (Yeh et al., 2014). Distinct changes in spatiotemporal gait parameters, such as slower gait and increased gait variability, occur during aging. Increased gait variability, specifically with mediolateral perturbations, poses a particular challenge for elderly adults and is linked with increased risk of falls. Virtual reality training has shown promising effects on improving balance and gait (Osoba et al., 2019).

Postural balance, body-orienting reflexes, muscle strength and tone resistance, and height of stepping all decrease with age and make the elderly more susceptible to falls (Ahn et al., 2019). Research in fall-prevention has increased during the past 10–15 years. A number of programs (e.g., risk factor reduction, exercise, environmental modification and education) were tested, and a meta-analysis identified the effectiveness of several approaches (Bhasin et al., 2020). Exercise programs improve muscle strength, endurance, and body mechanics, and reduce falls (Sherrington et al., 2019). There is a need to create intervention programs to improve balance and muscle strength that can reduce the number of falls while also restoring quality of life in seniors (Weber et al., 2018). Several studies investigated the effects of exercise using visual biofeedback as an intervention tool that can be used in balance rehabilitation (Alhasan et al., 2017).

Combined balance and strength training, in which visual feedback is provided, is a treatment program that fully engages the patient during rehabilitation. Laboratory-based devices such as force plates are used for balance and gait assessments and therefore, are informative of biomechanical changes directly affecting an individual’s static and dynamic balance (Scorza et al., 2018). Exercise programs using a force plate allow patients to check their positions and the location of the center of gravity during postural changes in real time, enabling patients to perceive postural information and use it to control and maintain their posture (Lee and Sun, 2018). A synthesize evidence from systematic reviews reported that combined balance and strength training are effective methods for improving postural balance following stroke (Arienti et al., 2019). Additionally, randomized controlled trials (RCT) comparing combined balance and strength training programs with conventional physical therapy reported benefits for patients treated with augmented visual biofeedback, for example on its effects on closed eye posturographic measures (Ghomashchi, 2014), persistent improvements in dynamic stability measures and functional scores (Srivastava et al., 2009), reductions in asymmetry and sway in task performance, reductions in postural sway measures and improvements in stance symmetry and daily living activities (Hwang et al., 2017).

Visual feedback approaches in rehabilitation have been studied in a variety of patient populations including those with stroke (Ghomashchi, 2014; Hwang et al., 2017), Parkinson’s disease (Lang et al., 2019), cerebral palsy (Widener et al., 2020), vestibular deficits (Sienko et al., 2017), diabetes (Melese et al., 2020), and upper-limb (Antfolk et al., 2013) and lower-limb amputees (Sharma et al., 2014; Leineweber et al., 2016). Prata and Scheicher (2014) reported that training program consisting of muscle strengthening and balance is the most promising intervention for reducing the number of falls and the fear of falling. However, there are only a few studies that have investigated the effects of combined balance and strength training On postural balance and mobility function, with visual feedback, to prevent falls in women (Sihvonen et al., 2004a,b; Hatzitaki et al., 2009). For example, Hatzitaki et al. (2009) investigated the effects of 4 weeks (3 sessions a week for 25 min per session) of two direction-specific, visually guided weight-shifting training protocols (anterior/posterior vs. medio/lateral directions) on standing balance of healthy older women. There was a greater improvement of static balance control when using anterior/posterior direction compared to medio/lateral direction. A study by Sihvonen et al. (2004a) reported significant improvements of balance control in frail older women after 4-weeks of visual feedback-based balance training. Moreover, another study by Sihvonen et al. (2004b) reported that individualized visual feedback-based balance training could be a promising method for fall prevention among frail older women. It is important to note that the studies by Hatzitaki et al. (2009) and Sihvonen et al. (2004a, b) lasted only 4-weeks where the control groups did not receive other training or rehabilitation programs, making it difficult to assess the effectiveness of the experimental training program. Therefore, more information is needed on the effects of visual biofeedback training on postural balance and mobility function. The positive effects of combined balance and strength training as an adjunct to conventional physical therapy exercises in women with a history of falls is unclear, and evidence for the effectiveness of this treatment program is needed prior to using it in patients.

Consequently, our study investigated the effects of combined balance and strength training on measures of balance and muscle strength in older women with a history of fall.

Based on previous studies (Sihvonen et al., 2004a,b; Hatzitaki et al., 2009), we hypothesized that a combined balance and strength training program could be a useful preventive strategy for women at risk of falls by improving transfer and control of participants center of gravity (COG) in weight-bearing squat (WBS), sit to stand (STS), shift their COG laterally, forward, and backward to maintain static and dynamic balance.

## Materials and Methods

### Participants

**FIGURE 1:**
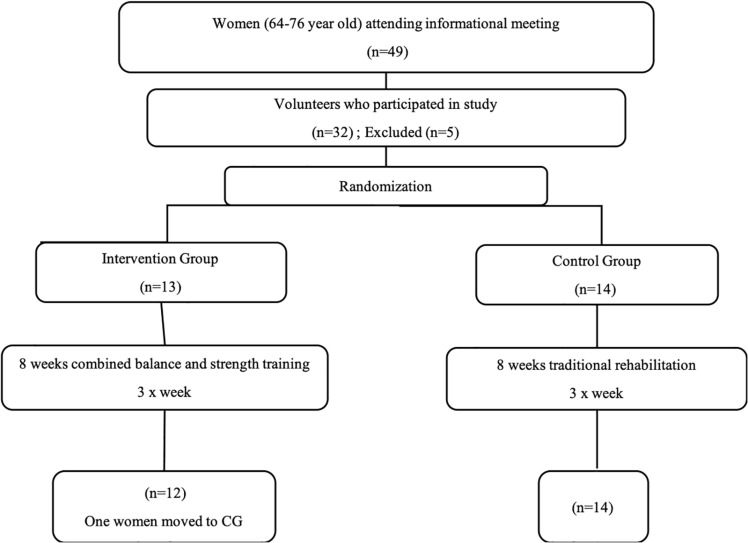
Flow chart of the study design.

Twenty-seven older women aged between 65 to 75 years with a history of falls were recruited from an external consultation with Physical Medicine and Functional Rehabilitation of the National Institute of Orthopedics Mohamed Kassab, Tunis, Tunisia. Participants were randomly divided into two groups, the intervention group (IG, *n* = 12; age = 70.4 ± 3.17 years; height = 166.3 ± 5.2 cm; body mass = 76.6 ± 7.2 kg) and the active control group (CG, *n* = 15; age = 72.0 ± 3.5 years; height = 162.0 ± 5.5 cm; body mass = 78.5 ± 7.6 kg) (Figure 1). Each participant provided written informed consent and underwent a medical history and medication review. The inclusion criteria for the study were: (1) able to walk more than 10 meters, either without or with assisting devices such as orthotics, a walker or a cane; (2) no symptoms of any severe neurological (vestibular, visual, and proprioception) function or orthopedic abnormalities (spine and foot); (3) a Mini Mental State Examination Test (MMSE) score higher than 24 points; (4) no pain (lumbar, knees or feet) of intensity greater than 4/10 evaluated by a visual analog scale and (5) able to read the words on a monitor 60 cm away at eye level (Rempel et al., 2007). Participants had to be at least 60 years of age, have a body mass index (BMI) below 30 kg/m^2^ and have no prior history of pharmacologic treatment for osteoporosis (Kanis et al., 2008). All participants had normal ranges of motion in the neck and had no decreased range of motion in the lower extremities. Limb dominance was determined according to the lateral preference inventory for footedness (Stanley, 1993). For this purpose, 4 questions from the lateral preference inventory were used which include (i) with which foot would you kick a ball to hit a target?, (ii) if you want to pick up a pebble with your toes, which foot would you use?, (iii) which foot would you use to step on a bug?, and (iv) if you had to step up onto a chair, which foot would you place on the chair first?

The study was conducted according to the latest version of the Declaration of Helsinki and the protocol was approved by the Ethics Committee of the National Institute of Orthopedics “M.T. Kassab” Tunisia.

The history of falls in the preceding 6 months prior to study entry was obtained by interviewing and using the performance-oriented mobility assessment test (POMA). A total score of 19 or less indicates a high risk for falling and a score between 19 and 24 indicates a moderate risk of falls (Yüçel et al., 2012). Based on the total scores obtained in the POMA test, the total score of the participants in our study was <19: Gait score = 6.33 ± 1.49/12; Balance score = 8.42 ± 1.92/16; Total score (Gait + Balance) = 14.32 ± 2.92/28.

### Measurements

The participants were tested for the assessment of muscle strength (weight-bearing squat [WBS] by measuring the percentage of body mass borne by each leg at different knee flexions [0°, 30°, 60°, and 90°], sit-to-stand test [STS]), and balance before and after the 12 week training program. Balance tests used the modified clinical test of sensory interaction (mCTSIB) with eyes closed (EC) and opened (EO) on stable (firm) and unstable (foam) surfaces, as well as spatial parameters of gait such as step width and length (cm) and walking speed (cm/s).

### Functional Test: The Performance-Oriented Mobility Assessment Test (POMA)

We used the original version of the 16-item POMA test, with a total score of 28 that includes two subscales of balance (9 items) and gait (7 items) (Jahantabi-Nejad and Azad, 2019).

The POMA is a clinical tool for evaluating balance and gait and is commonly used in different settings with some level of experience; it is simply implemented by using a standard chair and a chronometer. The POMA includes two subscales of balance and gait. The balance subscale has a maximum score of 16 and includes the following items: 1-sitting balance; 2-arise; 3-attempt to arise; 4-immediate standing balance (first 5 s); 5-standing balance; 6-nudged (participant at a position with feet as close together as possible, examiner pushes lightly on the individual’s sternum with the palm of hand three times); 7-the same as item 6 but with eyes closed; 8-turning 360 degrees; 9-sitting down. The gait subscale has a maximum score of 12 and includes the following items: 1-initiation of gait (immediately or after saying to “go”); 2-step length and height; 3-step symmetry; 4-step continuity; 5-path deviation; 6-trunk stability; 7-walking stance (heels apart/heels almost touching while walking). Some items are scored as binary (0 = cannot perform, 1 = can perform) while the others are scored as 0, 1, and 2 (0 = abnormal, 1 = adaptive, and 2 = normal). Participants were allowed to rest between items if needed, and were allowed to have one pilot attempt to familiarize them with the procedure. The POMA had a total score between 0 and 28, with low scores indicating an increased risk of falling (Tinetti, 1986).

### Instrumental Tests

Before and after 12 weeks of training, participants were tested for the assessment of muscle strength (weight-bearing squat [WBS] by measuring the percentage of body mass borne by each leg at different knee flexions [0°, 30°, 60°, and 90°], sit-to-stand test [STS]), and balance. Balance tests comprised the modified clinical test of sensory interaction (mCTSIB) with eyes closed (EC) and opened (EO) on stable (firm) and unstable (foam) surfaces as well as spatial parameters of gait such as step width and length (cm) and walking speed (cm/s). All tests were performed on a force plate [NeuroCom^®^ Balance Master^®^ (NeuroCom International; Software Version 7 NeuroCom^®^ International, Inc©. 2008)].

### Weight-Bearing/Squat

This test provides an objective measure of the patient’s ability to perform squats while maintaining equal body mass on both extremities at different degrees of knee flexion. The percentage of body mass borne by each leg (left and right body mass) was measured while standing at 0° (erect position), 30°, 60°, and 90° of knee flexion (Lim and Lee, 2012). The percentage of mass supported by the leg was expressed numerically, and parameters were measured separately for each leg and each position.

### The Sit to Stand (STS) Test

Dynamic balance was evaluated by quantifying the participant’s ability to rise from a stable surface positioned on the wooden platform of the system. This task included changing the center of gravity forward in the initial position and on the support base (feet) followed by trunk extension in the upright position. Two important aspects worth noting in this test are: (i) the initial sway velocity indicates the degree of sway that the patient presents immediately upon standing, and (ii) the percentage (%) left/right symmetry indicates how much weight is borne by each extremity during the sit to stand motion (Lim and Lee, 2012). Data from the sit to stand test included time to transfer weight over the center of base of support (s), rising power (%), and speed of sway of COG (deg/s).

The STS was used to assess balance point movement control during the change from a sitting to a standing position. Participants were instructed to stand up from the sitting position and to maintain a standing position (10 s in the control group and 30 s in the experimental group). The participants sat on a wooden bench with felt pads, from 100° in the knees and 90° in the hip, with the body mass equally distributed to both legs. The parameters observed in this test were the Rising Index (RI, measured as a percentage) which represents the force exerted by the legs at the standing-up stage and the COG Sway Velocity (CSV), which measures the control of the balance point above the support base during the transfer, and expressed in degrees per second (deg/s). The participants repeated the test three times and the best performance served as their score (Trajkov et al., 2015).

### Modified Clinical Test of Sensory Interaction on Balance (mCTSIB)

This test analyzed static balance by quantifying the speed of oscillation of COG (deg/sec) while the individual was standing on the platform under different conditions: eyes open on a firm surface (firm EO); eyes closed on a firm surface (firm EC); eyes opened unstable surface (foam EO); closed eyes on an unstable surface (foam EC) (Freeman et al., 2018). We used a foam block (50 cm in length and breadth, 20 cm in height and a density of 0.5 kg/m^3^) that was integrated with the Balance Master System (NeuroCom^®^ Balance Master^®^, NeuroCom International; Software Version 7 NeuroCom^®^ International, Inc^TM^. 2008) to analyze balance on an unstable surface. A mask for blindfolding was used for closed-eye conditions.

### Walk Across (WA)

The WA is a performance test that quantifies the patient’s steady state gait while walking across a force plate. The WA enhances observational testing of gait by measuring the average width and length of the patient’s steps on the force plate, the symmetry of left and right leg step lengths, and the patient’s gait speed across the force plate.

Participants walked at a comfortable and preferred pace across the long surface (45 cm × 150 cm) of the plate and the scores of three trials were averaged. Spatial parameters of gait were measured on the force plate (step width and length [cm]) and walking speed (cm/s) (Yang et al., 2011).

All measurements were clinically feasible and could be safely administered for older people (least 65 year of age). The ICC values were superior and the coefficient of variations (CVs) were less than 11% in all clinical balance and mobility measures. Most balance and mobility measures obtained using the NeuroCom^TM^ force plate (modified Clinical Test of Sensory Interaction on Balance, Walk Across (step width, step length parameters), and Sit to Stand [rising index parameter]) had excellent relative reliability (ICC_3_,_1_ ranging from 0.75 to 0.91). Values ICCs (0.40–0.75) were fair to good for the weight bearing squat test (Suttanon et al., 2011).

### Physical Therapy Training

The physical therapy protocol for the active control (CG) and training groups (EG) included physical therapy techniques aimed at improving muscle strength, range of motion, balance, and mobility. The exercise program sessions were tailored to each participant’s needs and individuals in both groups were encouraged during their sessions.

#### The Active Control Group

The active control group received only physical therapy training, which were provided 3 times per week for 60-min per session. This intervention included mat activities (stretching and muscle strengthening), weight bearing or shifting and standing lower-extremity exercise and balance activities such as rocker-board and unilateral stance activities, tandem stance activities and sitting with eyes opened and eyes closed, as appropriate but without visual feedback. The administered physical intervention consisted of exercises in which the participants were asked to increase their step length, step height, the mobility of the cervical rachis, and ocular mobility in order to develop muscle strength, flexibility, static balance with eyes opened and eyes closed. The difficulty level was increased during each training week by increasing the number of repetitions and tasks required (Thomas et al., 2019). The gait training exercises of the active control group are meant to help strengthen muscles or improve stability. These activities include stepping over objects, balance activities such as lifting the legs and unilateral stance, and tandem walk tasks.

#### The Training Group

For the training group, the physical therapy intervention was replaced by a specific training program implemented with force plates for 60 min per session, 3 times per week for 8 weeks. A screen was positioned in front of the participants during balance activities to provide continuous visual feedback. Each session started with a short (15 min) warm-up (walking in place, one-leg standing, weight shifting, lunges, arm and neck movements) followed by the scheduled exercises. The program training also incorporated weight-bearing squats, sit to stand, limits of stability and rhythmic weight shifting to improve balance and strength capacities. The use of force plate training protocols included a variety of task goals requiring propulsive movements of the body’s COG, high-velocity movements and movements on unstable surfaces. The plan of care focused on improving functional limitations such as balance, transfers and gait. Each training session lasted 1 h.

The training protocol used is shown in Table 1. The training program also included mat activities, weight bearing or shifting and standing lower-extremity exercises and balance activities such as unilateral stance activities and tandem stance. Training protocols were individualized and progressed by increasing the limits of stability and the pace (time allowed per weight shift) to challenge the participants weight-shifting abilities as their balance improved during training.

**TABLE 1:** Program of intervention using the force plate.

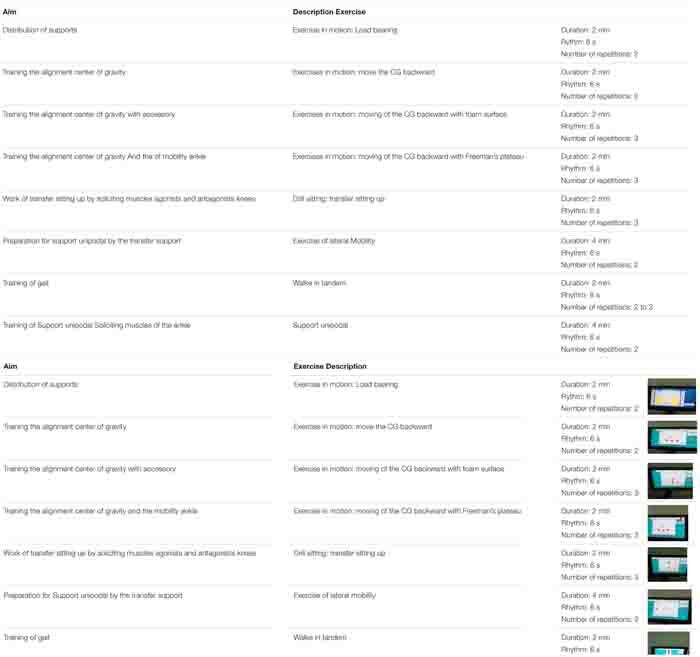

The limit of stability of a person is the distance they are willing and able to move without losing balance and taking a step, and is termed the perceived limit of stability (Coelho et al., 2017). The goal of most tests measuring limits of stability (LOS) is to examine an individual’s ability to control their center of gravity (COG). The LOS training protocols required the patient to lean toward 8 targets that were in forward, backward, and lateral directions. The tasks could be adapted with optional system accessories such as the Freeman tray, inclined access, obstacles or foam cushions (Table 1).

### Statistical Analyses

An *a priori* power analysis using G^∗^Power software (Faul et al., 2007) was conducted with the following input parameters to obtain medium-sized group by time interactions: Cohen’s f effect size (i.e., ES = 0.52), type I error (i.e., 0.01), type II error (i.e., 0.9), number of groups (i.e., 2), and number of measurements (i.e., 2). The inclusion of a medium effect size was based on a similar study conducted by Lacroix et al. (2016) who investigated the impact of combined balance and strength training on a repeated sit to stand task in healthy older adults. Our analysis revealed a total sample size of 22 (i.e., 11 participants per group). We enrolled more participants (*N* = 27) to make sure that we would not fall below this number due to potential drop-outs (e.g., injuries, personal reasons).

Data are presented as means ± standard deviations (M ± SD). After normality of data distribution was confirmed using the Shapiro–Wilk test, differences between and within groups were calculated using a two-way analysis of variance (ANOVA) for repeated measures. If a statistically significant interaction effect was detected, Bonferroni corrected *post hoc* tests were calculated. Effect sizes (ES) for main effects of group, time, and group by time interactions were taken from ANOVA output by converting partial Eta-squared to Cohen’s d (Cohen, 1988). Within-group ES were computed according to Morris using the following equation: ES = (mean pre-post change from IG – mean pre-post change from CG)/pooled pretest SD (Morris, 2008). In accordance with Hopkins et al. (2009), ES were considered trivial (<0.2), small (0.2–0.6), moderate (0.6–1.2), large (1.2–2.0), and very large (2.0–4.0). The level of significance was set at *p* < 0.05. All statistical analyses were computed using SPSS for Windows, version 16.0 (SPSS Inc., United States).

## Results

All participants from the two experimental groups (IG and CG) completed the study according to the study design and methodology. No injuries related to training or testing occurred during the experimental period. During the 8-weeks intervention period, attendance rates was 94.7% for IG and 92.3% for CG. Results from the lateral preference inventory for footedness revealed that all participants were right dominant. There were no differences in participant characteristics at baseline between the IG and CG (Tables 2–5).

**TABLE 2:** The percentage distribution of body weight according to the different degrees of flexion of the knees for the experimental group (EG) and control group (CG).

			**EG**		**CG**		***p*-values (effect size)**		
			**Pre**	**Post**	**Pre**	**Post**	**Time**	**Group**	**Group × Time**
***Weight-Bearing/Squat (%)***	**Left leg**	**0°**	55.831.8	52.835.46	47.24.16	49.862.13	0.863 (0.002)	0.002(0.745)*	0.001(0.587)*
		**30°**	57.164.74	52.45.17	46.933.84	50.733.08	0.66 (0.014)	0.001(0.716)*	0.002(0.51)*
		**60°**	58.257.55	50.666.92	46.93.41	50.82.14	0.224 (0.104)	0.002(0.616)*	0.001(0.761)*
		**90°**	58.412.01	51.54.8	48.83.4	51.062.01	0.041(0.265)*	0.001(0.527)*	0.002(0.524)*
	**Right leg**	**0°**	44.161.8	47.165.46	534.61	50.252.22	0.919 (0.001)	0.003(0.722)*	0.004(0.54)*
		**30°**	42.834.74	47.585.17	53.463.95	49.233.32	0.834 (0.004)	0.001(0.702)*	0.004(0.505)*
		**60°**	41.758.15	49.334.32	533.67	49.232.31	0.282 (0.096)	0.002(0.58)*	0.001(0.732)*
		**90°**	41.587.79	48.55.41	50.583.55	48.912.23	0.066 (0.274)	0.011(0.443)*	0.013(0.441)*

Group-specific data are expressed as means and standard deviations (SD). The table illustrates main effects of group, time, and group by time interactions (*p < 0.05).

**TABLE 3:** Center of gravity oscillation velocities under different experimental conditions of the modified clinical test of sensory interaction on balance (mCTSIB) for the experimental (EG) and the control group (CG).

		**CG**		**EG**		***p*-values (effect size)**		
		**Pre**	**Post**	**Pre**	**Post**	**Time**	**Group**	**Group × Time**
**Center of gravity oscillation speed (°/s)**	**Firm EO**	0.340.20	0.450.47	0.40.26	0.360.25	0.242 (0.12)	0.730 (0.011)	0.021(0.362)*
	**Firm EC**	0.250.09	0.320.13	0.440.20	0.360.10	0.846 (0.004)	0.006(0.518)*	0.029(0.362)*
	**Foam EO**	0.420.11	0.470.24	0.960.26	0.780.20	0.63 (0.022)	0.001(0.765)*	0.006(0.518)*
	**Foam EC**	0.520.15	0.530.24	2.180.75	1.660.52	0.092 (0.236)	0.002(0.914)*	0.052 (0.302)

Group-specific data are expressed as means and standard deviations (SD). The table illustrates main effects of group, time, and group by time interactions (*p < 0.05). Firm, stable surface; Foam, unstable surface; EO, eyes open; EC, eyes closed.

**TABLE 4:** Transfer time survey index and transfer speed in the sit to stand test (STS) for the experimental group (EG) and control group (CG).

	**CG**		**EG**		***p*-values (effect size)**		
	**Pre**	**Post**	**Pre**	**Post**	**Time**	**Group**	**Group × Time**
**Transfer time (s)**	1.110.38	0.910.45	0.850.91	0.840.19	0.493 (0.048)	0.277 (0.117)	0.602 (0.028)
**Transfer speed (°/s)**	2.700.95	3.520.96	4.142.50	4.650.60	0.203 (0.156)	0.008(0.526)*	0.73 (0.012)
**Rising index (%)**	34.6610.3	40.426.34	43.8722.12	37.6311.56	0.974 (0.001)	0.484 (0.05)	0.107 (0.239)

Group-specific data are expressed as means and standard deviations (SD). The table illustrates main effects of group, time, and group by time interactions (*p < 0.05).

**TABLE 5:** Gait measures for the experimental group (EG) and control group (CG).

	**CG**		**EG**		***p*-values (effect size)**		
	**Pre**	**Post**	**Pre**	**Post**	**Time**	**Group**	**Group × Time**
**Step width (cm)**	17.223.13	17.062.96	15.592.85	16.042.98	0.905 (0.002)	0.042(0.353)*	0.704 (0.015)
**Step length (cm)**	59.439.93	66.9213.55	40.1712.33	72.6126.75	0.003(0.615)*	0.083 (0.270)	0.066 (0.362)
**Walking speed (cm/s)**	76.7612.55	76.5210.4	43.8512.05	54.1715.71	0.173 (0.177)	0.001(0.742)*	0.139 (0.205)

Group-specific data are expressed as means and standard deviations (SD). The table illustrates main effects of group, time, and group by time interactions (*p < 0.05).

The group × time interactions for the percentage distribution of body weight during the weight bearing squat for the different degrees of flexion of the knees are shown in Table 2. There were significant interactions for both legs and for all degrees of flexion (0.0001 < *p* < 0.013, 0.441 < *d* < 0.761). *Post hoc* tests revealed significant pre-to-post improvements for both legs and for all degrees of flexion in the IG group (0.0001 < *p* < 0.002, 0.697 < *d* < 1.875).

The group × time interactions for the COG oscillation velocities under different conditions (firm EO, foam EO, firm EC, and foam EC) of the mCTSIB are shown in Table 3. There were significant interactions for the four conditions (firm EO, foam EO, firm EC, and foam EC) (0.006 < *p* < 0.029; 0.302 < *d* < 0.518). *Post hoc* tests revealed significant pre-to-post improvements for IG for both legs and for all degrees of oscillations (0.0001 < *p* < 0.004, 0.753 < *d* < 2.097).

The group × time interactions for the parameters of transfer time survey index and transfer speed in the sit to stand test (STS) are presented in Table 4. There were no significant interactions for transfer time (*p* = 0.602; *d* = 0.028), transfer speed (*p* = 0.730; *d* = 0.012), and rising index (*p* = 0.107; *d* = 0.239).

The group × time interactions for gait measures are shown in Table 5. There were no significant interactions for step width (*p* = 0.602; *d* = 0.028), step length (*p* = 0.73; *d* = 0.012), and walking speed (*p* = 0.107; *d* = 0.239).

## Discussion

We investigated the effects of a combined balance and strength training program on balance and muscle strength in older women and compared this to an active control group receiving only conventional physical therapy. Our results show that combined balance and strength training using a visual feedback-based force platform improved displacement, and COG velocity mobility in the WBS, STS and improved the participant’s ability to shift their COG laterally, forward and backward to maintain static and dynamic balance. Furthermore, the 8-weeks strength and balance training program resulted in significant group × time interactions for the percentage distribution of body weight between legs at different conditions of knee flexion (0°, 30°, 60°, and 90°) and also decreased the sway oscillation under four conditions (firm EO, foam EO, firm EC, and foam EC) in the IG. These results suggest that a combined balance and strength training program including visual biofeedback improves static balance in older women, and will likely reduce falls.

Asymmetries were found in the study participants. Thus, it has previously been postulated that asymmetry between limbs may be predictive of future falls given that risk of falling increased with asymmetry. For example, Skelton et al. (2005) indicated that asymmetry is prevalent in women aged 65 and over, and associated with a history of falls. When participants performed an increased knee flexion during a weight bearing squat, asymmetry occurred in favor of the left leg (Roerdink et al., 2009). However, to maintain lateral balance, participants need to adjust their posture to avoid falling. Poor weight shifting abilities (Robinovitch et al., 2013) and deterioration in neuromuscular, musculoskeletal and sensory systems with aging means increased difficulties for older women to control standing symmetry (de Vries et al., 2014). Squat tests place a greater demand on the knee extensors and ankle plantar flexors in older adults. Clinicians may use this discriminate finding to more effectively target specific lower-extremity muscle groups when prescribing exercise programs for older adults. Weight-shift training improves the weight distribution during quiet standing and at different positions of knee flexion between the legs. The ability to transfer body weight from one leg to the other is an essential aspect of human locomotion and is frequently utilized during activities of daily living such as walking (Nakajima et al., 2013).

Postural biofeedback balance training for improving dynamic stability can be applied by weight shifting to selected targets displayed on a computer screen (Srivastava et al., 2009; Laroche et al., 2012). Combined balance and strength training improve the dynamic balance and sensory integration capabilities of older adults with a history of falls (Barzegari et al., 2019). Postural asymmetry necessitates higher energy expenditure and the use of particular control mechanisms (Genthon and Rougier, 2005). Many studies of the older report that strength training increases lower body muscle mass and strength, and it is reasonable to assume that stronger legs provide a more stable base of support (Paillard, 2017). A decline in lower extremity strength is associated with an increased risk of falls (de Souza Vale et al., 2009). Our results suggest improvements in static balance allows visual feedback to reduce fall risk in older adults (Villareal et al., 2011).

The asymmetry of weight distribution induces an asymmetry of sensory stimuli applied to load-sensitive sensory receptors (OTG, plantar skin receptors, neuromuscular spindles, and articular receptors) (Genthon and Rougier, 2005). Keeping an optimal balance requires a complex interaction between internal factors (proprioception, auditory, and visual senses) and muscular factors. These interactions have a reciprocal effect on the neural network and movement feedbacks. All factors involved in balance are affected by the aging process and leads to falling (Barzegari et al., 2019). Falling is associated with a reduced sensitivity of such sensory receptors that monitor the body’s orientation (Goble et al., 2009). Impairments in sensory organization for balance can be measured by increases in body sway when sensory information for balance is altered (Horak et al., 2009). The CTSIB test is considered the gold standard for quantifying postural sway and fall risk in the older (Buatois et al., 2006). This test measures the time that a patient can remain standing in a particular position and serves as an indirect indicator of motion oscillation but is insensitive to subtle changes or slight deficits (Freeman et al., 2018). The participants in our study experienced increases of sway velocity of COG on a firm surface (when their eyes were closed) and also on a foam surface (eyes opened and closed). In the intervention group, this latter finding was likely related to a reduced sensitivity due to an overreliance on visual feedback (Yeh et al., 2014) that can disrupt balance when visual inputs are altered or unreliable (Jeka et al., 2006; O’Connor et al., 2008). Our intervention program reduced sway oscillations of COG on a firm surface (with EO and EC) and on a foam surface (EO and EC). These results suggest that older women may rely more on vision to correct postural errors (Yeh et al., 2014).

A frequent explanation for the decrease in postural sway is an augmented native sensory input, giving the user more information about body position with respect to gravity (Haggerty et al., 2012). Such a biofeedback signal helps to reduce the sway oscillation of trunk in visual and combined conditions, mostly during stance on a foam surface (Halická et al., 2014). It is likely that concentrating on additional vibrotactile information (along with visual and somatosensory feedback for balance control) probably also increases attention demands in the older. Such individuals reduce their postural sway by using visual and combined biofeedback during the stance on unstable foam surfaces; stance on foam alters proprioceptive inputs from the feet and a greater reliance on visual information (Dozza et al., 2005). Our study suggests that an unstable surface helped older people to maximally utilize additional sensory information.

Other interventions such as general strength and endurance programs were used concurrently with the force plate training programs. Improvements in strength and endurance could have played a role in improving the patient’s ability to complete functional tasks such as balance and transfers (from the sitting position) and in maintaining a standing position. We measured transfer time and speed, control of the balance point above the support base during the transfer, and 10 and 30 s afterward; the data were expressed in degrees per second (deg/s), and the rising index (RI, which represents the force exerted by the legs at the standing-up stage) measured as a percentage. Group × time interactions and *post hoc* tests indicated no improvements in the intervention group. The participants were instructed to walk along the entire length of the platform in the WA test, following their own rhythm and pace. There were no group × time interactions in our study, and *post hoc* tests indicated no improvements due to the intervention.

Static balance affects almost all activities of daily living. Therefore, static and dynamic balance training programs are important parts of rehabilitation. Visual information can compensate for the loss of somatosensory function and facilitate the control of motor regulation in the brain, so increasing the effectiveness of treatment (Clark et al., 2010; Schiavinato et al., 2010). Combined balance and strength training reduce asymmetry of body alignment and can be a more effective balance training method than auditory or tactile feedback (Ko et al., 2015). For persons with a history of falls, rehabilitation programs using foot force plates can improve symmetry while standing (Scorza et al., 2018). In addition, training with asymmetric distribution of body weight improves symmetry standing recovery in older patients (Cheng et al., 2001). Accordingly, combined balance and strength training can significantly improve standing balance and gait function in the older (Lakhani and Mansfield, 2015), while also improving sensory reliability, and delaying the transmission of feedback from the lower limbs (Hatzitaki et al., 2009).

Our study has a number of limitations that warrant discussion. We focused on muscle strength rather than on the rate of torque development. Generally, rate of torque development appears to be more important than maximal muscle strength for balance (Bento et al., 2010), and previous studies attempted to increase RTD and standing balance (Kobayashi et al., 2016; Ema et al., 2017). Moreover, we examined only women and a specific type of training. Therefore, the findings of our study may not be generalizable to other populations or training types. In addition, the duration of our training program and our sample size are limited.

## Conclusion

We report that combined balance and strength training with a visual feedback-based force platform improved the percentage distribution of body mass between legs at different conditions of knee flexion (0°, 30°, 60°, and 90°) and also decreased the sway oscillation on a firm surface (with eyes closed) and on a foam surface (eyes opened and closed) in the IG. Hence, training based on visual biofeedback reduced static postural sway in older women, although longer training periods may be required for correcting dynamic balance. A combined balance and strength training program for women with a history of falls improved transfers and control of COG and reduced postural sway and improved weight-shifting ability and balance when standing. We are among the first to show that computerized balance training with visual feedback in older women allows users to make changes in motion, allowing them to develop strategies to restore mobility function. Overall, the greater positive effect of training in the standing balance tests, compared with dynamic balance, suggests that balance training exercises including lateral, forward, and backward exercises improved static balance to a greater extent in older women.

### Practical Applications

To conclude, combined balance and strength training improved standing balance in older women. This program resulted in reductions in static postural sway in older women, although a longer training period may be required for correcting dynamic balance. It is important to optimize and customize biofeedback devices for each user, especially for older persons and for those needing to correct body movements for daily activities. An important benefit of visual feedback is the ability to provide real-time, continuous feedback to reinforce physiotherapy goals. The evidence to support the use of biofeedback in rehabilitation appears promising for the perception of spatial information that can reduce the incidence of falls in frail older women.

## Data Availability Statement

The raw data supporting the conclusion of this article will be made available by the authors, without undue reservation.

## Ethics Statement

The studies involving human participants were reviewed and approved by the study was conducted according to the latest version of the Declaration of Helsinki and the protocol was approved by the Ethics Committee of the National Institute of Orthopedics “M.T. Kassab” Tunisia. The patients/participants provided their written informed consent to participate in this study.

## Author Contributions

SZ, HZ, HF, and ABMZ conceived and designed the study. SZ and HF conducted the experiment. CD, ABMZ, and HZ analyzed the data. SZ, HZ, TP, AH, IL, UG, and ABMZ wrote the manuscript. All authors read and approved the manuscript.

## Conflict of Interest

The authors declare that the research was conducted in the absence of any commercial or financial relationships that could be construed as a potential conflict of interest.
